# Dietary Prevention of Atopic March in Pediatric Subjects With Cow's Milk Allergy

**DOI:** 10.3389/fped.2020.00440

**Published:** 2020-08-11

**Authors:** Laura Carucci, Rita Nocerino, Lorella Paparo, Carmen Di Scala, Roberto Berni Canani

**Affiliations:** ^1^Department of Translational Medical Science, University of Naples Federico II, Naples, Italy; ^2^ImmunoNutritionLab at the CEINGE Advanced Biotechnologies Research Center, University of Naples Federico II, Naples, Italy; ^3^European Laboratory for the Investigation of Food-Induced Diseases, University of Naples Federico II, Naples, Italy; ^4^Task Force for Microbiome Studies, University of Naples Federico II, Naples, Italy

**Keywords:** allergic march, food allergy, breast milk, infant formula, gut microbiota, epigenetics

## Abstract

Cow's milk allergy (CMA) is one of the most prevalent food allergies and the most expensive allergic diseases in the pediatric age. There is no cure for CMA, and actual disease management is based on strict avoidance of cow milk protein-containing foods, access to rescue medication, and use of substitutive formulas. Early-life CMA could be one of the first steps of the “allergic march” (AM), leading to the occurrence of other atopic manifestations later in the life, including asthma and oculorhinitis, with subsequent further increase of costs for health care systems and families of affected children. In the last years, diet is emerged as a relevant strategy to prevent allergic diseases through, at least in part, epigenetic modulation of immune system. We provide an overview of studies that investigate the potential role of different dietary strategies in preventing the AM in pediatric patients with CMA.

## Introduction

Affecting up to 3% of children worldwide, cow's milk allergy (CMA) is one of the earliest and most prevalent food allergies (FA) in the pediatric age. It is also responsible for the vast majority of food-induced anaphylaxis cases in the Italian pediatric population, with significant costs for the healthcare system and families, and it emerged as one of the most expensive allergic diseases (1–8).

**Figure 1:**
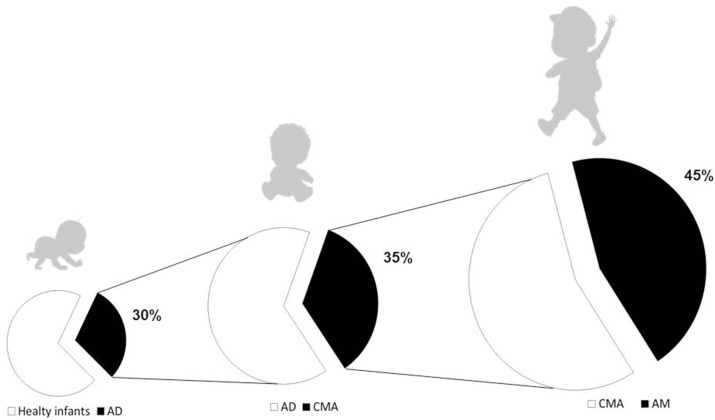
The atopic march in pediatric patients with cow's milk allergy. Atopic dermatitis (AD) is commonly considered the first step of the atopic march (AM), however, AD and cow's milk allergy (CMA) could co-exist, particularly in those with early onset, severe, and persistent atopic eczema. CMA affects about 1/3 of patients with AD. Data from several clinical studies demonstrate that up to 45% of children affected by CMA will develop other atopic manifestations later in the life, also after the immune tolerance acquisition to cow's milk proteins.

Although most subjects with CMA naturally outgrow it over time, studies evidence a wide range of ages and rates of resolution with an increased risk of persistence in recent decades, mainly due to negative gene–environment interaction leading to the breakdown of immune tolerance mechanisms (9–12). In addition, evidence suggests that early-life CMA could be one of the first steps of the “allergic march” (AM), leading to the occurrence of other allergic disorders during childhood. Indeed, the occurrence of allergic sensitization in these children increases the risk of later developing asthma and allergic oculorhinitis (AR), in particular when sensitization occurs along with atopic dermatitis (AD) (13–15). Figure 1 depicts the natural history of AM in CMA children. According to data from several clinical studies, up to 45% of CMA children develop other atopic manifestations later in the life, also after the immune tolerance acquisition to cow milk proteins (3, 5, 16–18). The development of AM is driven by genetic predisposition, but environmental factors may play a key role in its clinical expression. Indeed, as shown by longitudinal studies, only a minority of children follow the classic pathway of AM (starting from AD and followed by sequential development of FA, asthma, and AR) (19, 20). Earlier recognition of at-risk infants, regardless of CMA temporal appearance, allows fielding effective strategies to limit the occurrence of other atopic manifestations later in the life.

There is no cure for CMA, and actual disease management is based on strict avoidance of cow's milk protein-containing foods, access to rescue medication, and use of substitutive formulas (21–25).

Due to the increasing prevalence, persistence, and risk for developing other atopic manifestations in children with CMA, preventive strategies are highly advocated. In the last years, diet is emerging as a relevant strategy to prevent allergic diseases through the active modulation of the immune system (26). This review is focused on the potential role of different dietary strategies in preventing the AM in pediatric patients with CMA.

## The Potential of Breastfeeding

Breastfeeding is the best dietary strategy for newborn infants due to its optimal nutritional properties and several bioactive compounds that influence health status. Studies suggest a protective role on the onset of FA, asthma, and AD, both in low- and high-risk infants breastfed for at least 3–4 months (27–33). A WHO report suggests that allergic diseases are lower in exclusively breastfed compared to non-breastfed infants (34). A reduction of about 4% in FA risk for every additional month of exclusive breastfeeding has also been estimated (35). Unfortunately, most available data on breastfeeding and allergic diseases are based on observational, retrospective, underpowered studies, and present several confounding factors, such as the inclusion of partially breastfed infants (36, 37). Another limiting aspect is that the protective mechanisms against FA and other atopic manifestations are still not completely characterized. Breast milk contains several potential protective factors against allergy. Some compounds could be able to exert an indirect effect on immune system through a modulation of infant gut microbioma (GM), whereas other components could exert a direct modulatory effect on the infant immune system toward a protection against allergic diseases (38, 39) (Table 1).

**Table 1:** Main immunomodulatory factors in human milk.

**Regulation of infant's immune system through a direct interaction with immune cells**	**Regulation of infant's immune system through a modulation of gut microbiome**
Cytokines	Lactoferrin
Bacterial DNA	Lisozyme
miRNAs	Secretory IgA
Short chain fatty acids	Human milk bacteria
Human milk oligosaccharides	Human milk oligosaccharides
Omega-3 fatty acids	

The GM is emerging as a pivotal regulator of immune tolerance development (6). Breastfeeding shapes infant GM, both by direct transition of the human milk bacteria (HMBs), and indirectly through milk compounds such as human milk oligosaccharides (HMOs), secretory IgA, and antimicrobial factors, which could impact bacterial growth and metabolism (40). Studies have suggested that breast milk owns unique microbiome, including beneficial commensal and potentially probiotic bacteria (41). HMBs can originate from maternal skin, newborn oral cavity, or mostly from the maternal gut (the “entero-mammary pathway”) and are influenced by mode of delivery, with a lower bacteria variety and abundance in cesarean compared to vaginal delivery (42, 43). Breast milk is considered the second source of microbes to infant GM, and it has been estimated that breastfed infants could receive from human milk microbiota up to 8 × 10^5^ bacteria daily (44). Considering the pivotal role of GM in influencing the infant immune system function against CMA (45), it is possible to hypothesize that HMBs could be an innovative target of intervention. Interestingly, it has been demonstrated that in the milk of allergic mothers the bifidobacteria counts were significantly lower than in the milk of non-allergic mothers (46).

Regarding breast milk non-microbial components, human milk oligosaccharides (HMOs) are a group of non-digestible carbohydrates that are able to regulate the immune system function in a direct or indirect way. The HMO composition in breast milk is influenced by environmental (such as maternal diet) and genetic factors, and a possible role in FA has been suggested (47). Recent studies reported an association between different genetically induced HMO composition and the development of CMA and FA (47, 48). Interestingly, one recent study highlighted the ability of specific HMOs, pulled from human milk, to induce the maturation of human monocyte-derived dendritic cells (DC) (moDC). The derived HMO moDC are able to promote T reg induction from native CD4^+^ T cells, with a final tolerogenic effect on the infant's immune system (49); but the best characterized HMO properties are related to the prebiotic modulation of early microbial gut colonization with bifidobacteria and *lactobacilli*, which are involved in the production of tolerogenic metabolites short-chain fatty acids (SCFA), in particular, butyrate (41, 50–54). Supporting this view, it has been reported that the GM of allergic infants lacks genes encoding key enzymes for HMO metabolization with the consequent impairment of butyrate production (55).

Butyrate may prevent allergy diseases though different ways, involving a regulation of the epithelial barrier (at skin, gut, and respiratory tract level), a direct effect on Th1/Th2 cytokine expression, and the activation of regulatory T cells (Tregs) (56–60). Many effects are mediated by the epigenetic modulation of gene expression, suggesting the possibility of a long-lasting regulatory effect on immune tolerance network (6).

The origin of butyrate in breast milk is still largely undefined. The mammalian gland is able to regulate the concentration of several macro- and micronutrients in human milk. Thus, it is possible to hypothesize that some mechanisms of regulation could modulate the butyrate content in human milk. However, recent evidence supports the hypothesis that, at least in part, human milk butyrate could be produced by the HMBs. The hypothesis of a pivotal contribution by mammalian gland/breast milk microbiota in butyrate production is supported by recent observations demonstrating the presence of potential butyrate-producer bugs (54, 61–65).

An example of a potential pathway in butyrate production in breast milk could be derived by HMO metabolization by selected bacteria, as recently demonstrated by others (62, 66).

Of note, increasing observations demonstrate the presence of significant butyrate concentrations in breast milk, ranging from 0.01 to >5.0 mM (67–70) (Table 2).

**Table 2:** Available data on butyrate concentrations in human milk.

**References**	**N° samples**	**N° mothers**	**Min value** **(mM)**	**Max value** **(mM)**	**Methods**
Maria et al. (68)	150	30	0.1	0.23	GC-MS utilizing a lipase assisted sample preparation (deuterated butyric acid (BA-D7) as an internal standard)
Schwab et al. (70)	19	19	1.36	5.7	HPLC-RI with external standard
Prentice et al. (69)	102	102	0.0	0.4	H-NMR and GC-MS
Dai et al. (67)	180	60	0.29	0.48	Methyl esterification of SCFAs and GC analysis

*GC-MS, gas chromatography-mass spectrometry; HPLC-RI, high performance liquid chromatography with refractive index detection; H-NMR, nuclear magnetic resonance spectroscopy; SCFAs, short chain fatty acids*.

In line with these data, our preliminary observation from 109 healthy mothers show a median butyrate concentration in mature human milk of 0.75 mM (range 0.16–1.97 mM) (58). Interestingly, several preclinical data show that this butyrate concentration is able to modulate several components of immune tolerance network mainly through epigenetic mechanisms (6, 56–60).

Altogether, these data strongly suggest the potential pivotal role of a modulation of breast milk composition for innovate preventive strategies against CMA and against the occurrence of AM in CMA children.

## The Potential of Formula Choice

The first evidence on the possible role of infant formulas in preventing AM in CMA infants was provided about 10 years ago. In a prospective cohort study of 119 children with IgE-mediated CMA, a multivariate analysis of risk factors for the occurrence of AM revealed that the use of an extensively hydrolyzed casein-based formula (EHCF) represented a protective factor for other allergic diseases, compared to other hypoallergenic formulas or soy-based formulas (OR 0.76; 95% CI: 0.149–0.945, *p* = 0.038) (71).

To our knowledge, to date, only one randomized controlled trial was performed to test the potential of a formula-based dietary intervention on AM prevention in CMA pediatric patients (5). In this prospective trial, a total of 220 infants with IgE-mediated CMA (67% males, median age 5.0 months) were randomized into two dietary groups: 110 subjects were placed on EHCF-based diet, and 110 children were placed on EHCF + probiotic *Lactobacillus rhamnosus* GG (LGG)-based diet. Patients were followed up for 36 months. In the complete case analysis (CCA), the absolute risk difference (ARD) for the occurrence of at least one atopic manifestation over 36 months was −0.23 (95% CI −0.36 to −0.10, *p* < 0.001). Even under the worst-case scenario, a difference in favor of EHCF+LGG was still detected. Using the CCA estimate of the ARD, the number needed to treat was 4 (95% CI 3–10) (5). These findings are consistent with those of recent studies revealing that the first-line approach with EHCF+LGG for CMA infants may slow down the AM, compared to infants treated with other formulas. A retrospective observational study on 211 subjects with CMA was conducted for new score validation for the risk of developing AM, using selected clinical and laboratory data (72). The authors found that the type of substitutive formula for CMA treatment may influence the natural history of these children. They divided the patients into five groups, based on formula composition: vegetable-based formulas (rice or soy), high-grade extensively hydrolyzed formula (EHF) for those in which >95% of peptides were 1,000 kDa, high-grade EHF plus LGG (EHF+LGG), low-grade EHF for those with a higher proportion of peptides (>1,000 kDa), or amino acid–based formulas. Authors found that the risk of AM occurrence decreased in those treated with high-grade EHF (OR 0.42; 95% CI 0.20–0.87, *p* = 0.02), and these results were stronger in patients treated with high-grade EHF+LGG (OR 0.30; 95% CI 0.09–0.98, *p* = 0.048). The authors concluded that the first-line approach with EHF may be beneficial to prevent the occurrence of AM, and LGG implementation strengthened this trend. They supposed that the hypoallergenic composition of this high-grade EHF and the GM may have helped to positively influence the immune tolerance network, decreasing the risk of developing AM (72). Similarly, in a recent retrospective cohort study of 940 infants with CMA, a binary logistic regression analysis showed that infants fed with extensively hydrolyzed whey formula (EHWF) had a significantly higher relative risk at 24 months of AD (OR: 3.438; 95% CI: 1.975–5.985; *p* < 0.001) and asthma (OR: 2.651; 95% CI: 1.242–5.660; *p* < 0.02) compared with those fed with EHCF+LGG. The authors concluded that the first-line therapeutic approach for newly diagnosed CMA children with EHCF+LGG, reducing the development of other allergic diseases later in life, may slow down the AM (73). Current guidelines provided by scientific societies (EAACI, DRACMA, NICE, ESPGHAN, NIAID, BSACI) strongly suggest avoiding unmodified animal milk proteins for CMA dietary treatment. In addition, there is no evidence supporting the potential role of such mammalian milks in preventing AM in FA patients (74). All available studies focused on the potential role of formulas in preventing AM are summarized in Table 3.

**Table 3:** The studies exploring the potential of formula choice in preventing atopic march in pediatric patients affected by cow's milk allergy.

**References**	**Study design/population**	**Age**	**Sample size**	**Intervention/duration**	**Outcomes**	**Results**
Berni Canani et al. (5)	Parallel-arm RCT/IgE-mediated CMA	1–12 months	*N* = 220 *I* = 110 *C* = 110	I = EHCF+ LGG C = EHCF; for 36 months	The occurrence of any atopic manifestation (eczema, urticaria, asthma, oculo-rhinitis) during the 36 months of the study.	The ARD of any atopic manifestation for EHCF+LGG vs. EHCF was: (1) −0.23 [95% CI −0.36 to −0.10, *p* < 0.001] at CCA; (2) −0.22 [95% CI −0.35 to −0.09, *p* < 0.001] at SA-EQS; (3) −0.33 [95% CI −0.45 to −0.21, *p* < 0.001] at SA-BCS; (4) −0.08 [95% CI −0.21 to 0.04, *p* = 0.5] at SA-WCS. The SA-EQS estimate was very similar to the CCA estimate. On absolute grounds, the SA-BCS was 10% higher and the SA-WCS was 15% lower than the CCA estimate. Even under the worst case scenario, a difference in favor of EHCF+LGG was still present (8%). Using the CCA estimate of the ARD, the NNT was 4 (95% CI 3 to 10).
Sánchez-Valverde et al. (71)	Observational cohort study/IgE-mediated CMA	4 ± 2.63 months	*N* = 119	More extensively hydrolyzed high grade hydrolysates (+EH/HGH), which are those in which >95% of peptides are of < 1,000 kDa, and less extensively hydrolyzed hydrolysates and soya milk formulas.	To evaluate factors that could predict development of atopic march in children with IgE-mediated CMA	Multivariate analysis of risk factor, for the occurrence of AM revealed that EHCF use represented a protective factor for other allergic diseases compared to other hypoallergenic formulas or soy-based formula (OR 0.76; 95% CI: 0.149–0.945, *p* = 0.038).
Gil et al. (72)	Retrospective observational cohort study/only IgE-mediated CMA	Mean age at diagnosis 5.07 ± 2.67 months Mean age at the end of follow up 14.41 ± 5.42 years	*N* = 211	Five groups, based on formula composition: vegetable-based formulas (rice or soy), high grade EHF in which >95% peptides were 1,000 kDa, high-grade EHF + LGG, low-grade EHF in which higher proportion of peptides > 1,000 kDa, or amino acid–based formulas.	To evaluate if a new scoring system could determine the risk of developing allergic march	The risk of AM occurrence decreased in those treated with high grade EHF (OR 0.42; 95% CI 0.20–0.87, *p* = 0.02), and these results were stronger in patients treated with high-grade EHF + LGG (OR 0.30; 95% CI 0.09–0.98, *p* = 0.048).
Guest and Fuller (73)	Retrospective cohort study/IgE- and non IgE-mediated CMA	Mean age I = 4.2 ± 2.7 months C = 5.4 ± 2.9 months	*N* = 940 *I* = 470 *C* = 470	I = EHCF+LGG C = EHWF	The occurrence of any allergic manifestations over a period of 24 months from the start of formula	Binary logistic regression analysis showed that infants fed with EHWF had a significant higher relative risk at 24 months of atopic dermatitis (OR: 3.438; 95% CI: 1.975–5.985; *p* < 0.001) and asthma (OR: 2.651; 95% CI: 1.242–5.660; *p* < 0.02) compared with those fed with EHCF+LGG.

*RCT, randomized controlled trial; N, total number of subjects; I, intervention; C, control; EHF, extensively hydrolyzed formula; EHCF, extensively hydrolyzed casein formula; LGG, Lactobacillus rhamnosus GG; EHWF, extensively hydrolyzed whey formula; eHF, extensively hydrolyzed formula; IgE, immunoglobulin E; CMA, cow's milk allergy; AM, atopic march; ARD, absolute risk difference; CCA, complete case analysis; SA-EQS, sensitivity analysis—equal worst outcome scenario; SA-BCS, sensitivity analysis—best case scenario; SA-WCS, sensitivity analysis-worst case scenario; NNT, Number needed to treat; 95% CI, 95% confidence interval; OR, odds ratio*.

## Potential Mechanisms of Action of Infant Formulas

**Figure 2:**
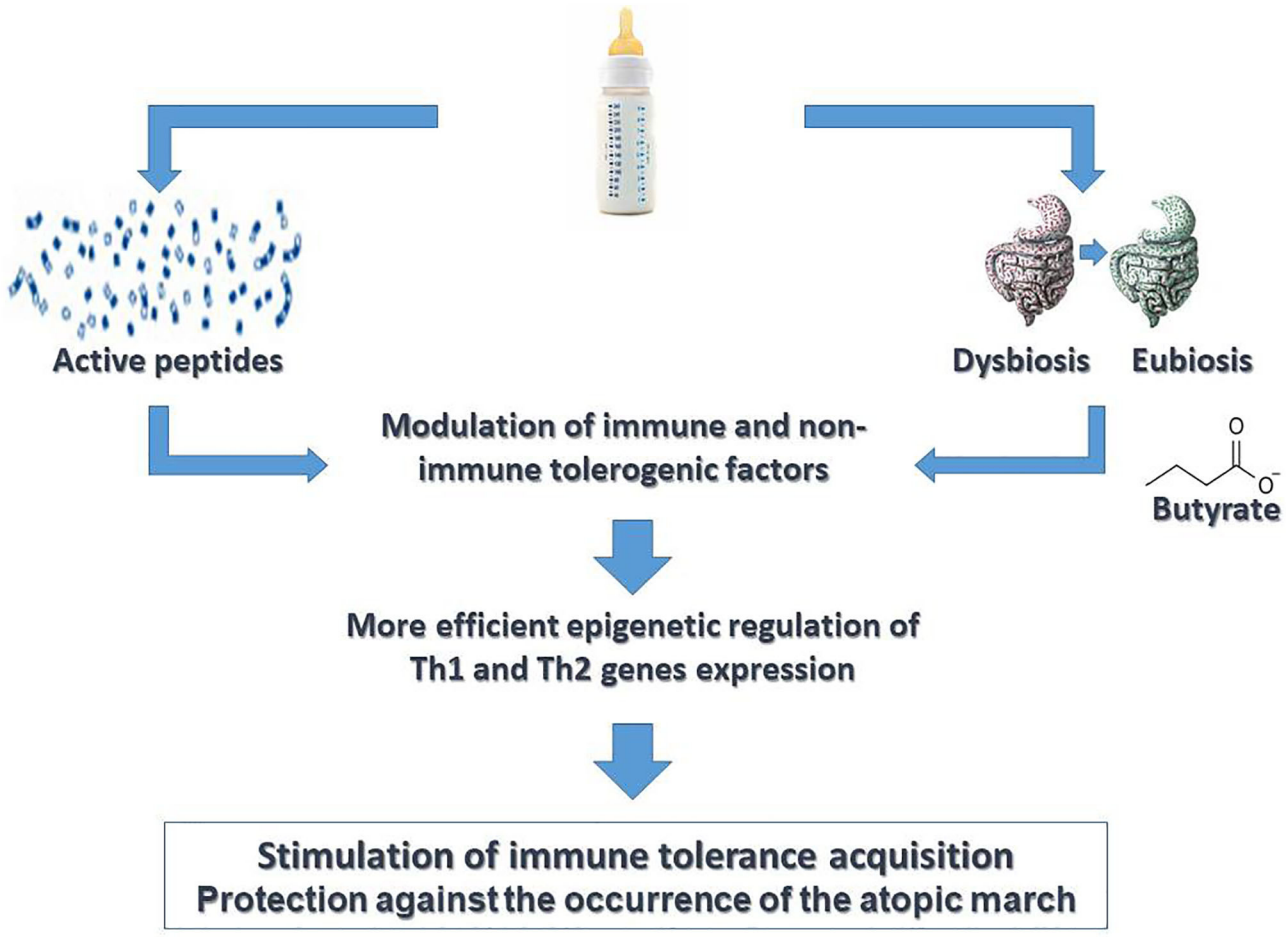
Active diet therapy in pediatric patients with cow's milk allergy. “Active diet therapy” means the possibility to influence the cow's milk allergy (CMA) disease course and to limit the occurrence of other atopic manifestations later in the life. Emerging evidence suggests the importance of formula choice for the management of CMA pediatric patients. It has been demonstrated that the use of extensively hydrolyzed casein formula (EHCF) containing the probiotic *L. rhamnosus* GG (LGG) could exert a modulation of immune tolerance network mediated by the activity of selected casein hydrolysis-derived peptides and by activity of LGG on gut microbiota structure and function leading to an increased production of the short chain fatty acid butyrate. Several non-immune (gut barrier integrity) and immune (cytokines, immune cells) tolerogenic factors are involved in such modulatory action. Many effects are mediated by epigenetic mechanisms. Altogether these mechanisms are able to stimulate a faster acquisition of immune tolerance to cow's milk peptides and to limit the occurrence of atopic march.

It has been suggested that selected milk protein hydrolysates used for CMA management may be able to not only avoid allergic symptoms in CMA infants due to the breakdown of IgE antigens but also play a role in immune system modulation, inducing tolerance and preventing allergic sensitization (75–79). These peptides are able to interact with TLR2 and TLR4, modulating cytokine release by epithelial and immune cells (80). It has also been demonstrated that specific peptides from casein hydrolysates, driving T cell switching from Th2 to Th1 or to Tregs subtype, could exert a protective effect for FA (77, 81). Animal studies have demonstrated that these peptides can suppress Th2 response through an IL-10 up regulation and IL-2 down-regulation (75). Moreover, the production of the tolerogenic cytokine IL-10 was higher in Jurkat T cells that underwent a casein hydrolysate stimulus (79). Preliminary data by our group suggest that formula choice is able to induce immune system modulation through epigenetic mechanisms in CMA infants (17, 82, 83); specifically, evidence suggests that EHCF+LGG is able to modulate GM, raising the abundance of selected genera (*Roseburia, Coprococcus*, and *Blautia*) with increased production of butyrate (16). A significant difference in DNA methylation of Th2 and Th1 cytokine (IL-4, IL-5, IL-10, and IFN-γ) genes and of FoxP3, the transcription factor that modulates the fate of Tregs, was observed in infants treated with EHCF+LGG who develop immune tolerance compared to children who received other formulas (82, 83). A DNA methylation status of all allergy-related genes in infants treated with EHCF+LGG was closer to that observed in healthy children. Analyzing the potential factors able to modulate DNA methylation status in tolerant children, the authors found that the variable that greatly influenced the DNA methylation status was EHCF+LGG formula use (82, 83). A longitudinal study, the EPICMA trial, compared the DNA methylation of FoxP3, Th1/Th2 cytokine genes, and allergy-related microRNAs (miRNAs) profile in IgE-mediated CMA infants taking EHCF+LGG compared to soy formula. This study demonstrated that treatment with EHCF+LGG is characterized by a more pronounced effect on FoxP3 demethylation compared to soy formula and by a higher methylation status of IL-4 and IL-5 and a lower methylation status of IL-10 and IFN-γ (17). Moreover, children treated with EHCF+LGG showed a selected miRNA expression toward a Th1-oriented response, leading to the activation of immune tolerance mechanisms (17). However, the impact of diet on epigenetic mechanisms may not only be direct but also mediated by the GM (84). So, the Diet-GM-Epigenetic axis creates a coherent picture that may be useful for developing potential strategies against AM in CMA children (Figure 2). Altogether, these data highlight the relevance of “immune-nutrition management” able to reduce disease duration and to protect against the occurrence of other atopic manifestations the CMA children.

## Conclusions

**Figure 3:**
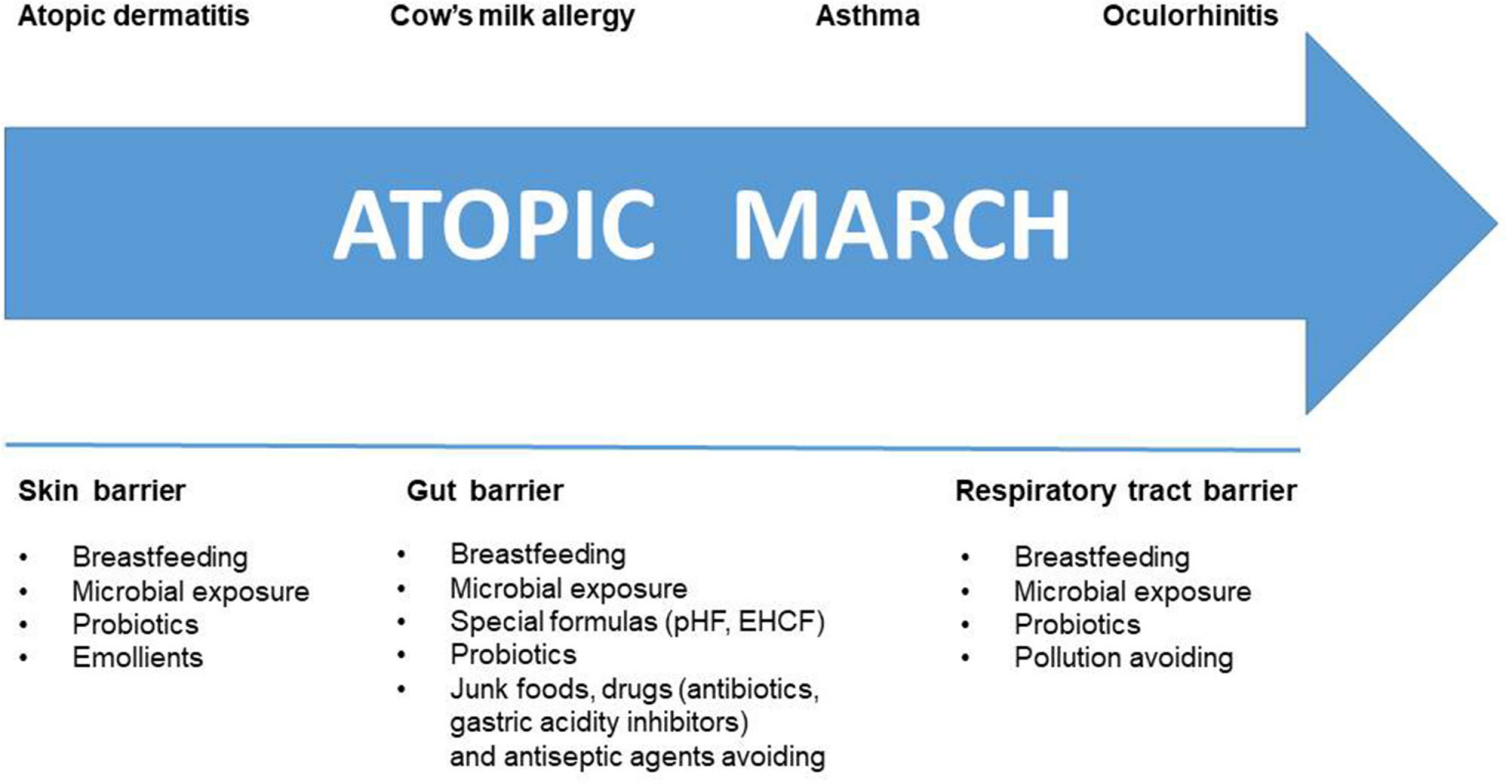
Halting the Atopic March. Several strategies are available to counteract step by step the atopic march. These strategies are targeting the skin, gut, and respiratory tract barrier.

During the last years, much has changed about AM knowledge. The actual strategies to halt the AM are depicted in Figure 3. Despite the lack of cure, novelties about CMA dietary management are moving from “passive” elimination diet to an “active diet-therapy” able to reduce disease duration and to protect against the occurrence of AM. The latter strategy is supported by better knowledge on the role of diet, breastfeeding, gut microbiome, and tolerogenic mechanisms. Thus, an active diet-therapy able to modulate the GM composition, restoring microbial equilibrium and optimal butyrate production, is a positive example of the potential of such strategy. The best nutritional choice for CMA infants is breastfeeding, but recent evidence suggests that breast milk composition could be influenced by environmental factors including maternal diet that could represent relevant target of intervention for preventive strategy against AM in CMA infants. If breastfeeding is not possible, evidence suggests that casein hydrolysate-based infant formula with the adjunction of the probiotic LGG could be able to stimulate immune tolerance acquisition and to reduce the incidence of AM in children with CMA.

## Author Contributions

RB designed and structured the review, wrote, and read the manuscript. LC and RN analyzed literature, wrote, and read the manuscript. LP and CD analyzed literature and read the manuscript. All authors listed have made a substantial, direct, and intellectual contribution to the work, and approved it for publication.

## Conflict of Interest

The Department of Translational Medical Science received research grants from Danone, Kraft Heinz, Humana, Mead Johnson Nutrition, Nestlè, and United Pharmaceutical. The authors have no other conflict of interests that are directly relevant to the content of this paper, which remains their sole responsibility.
